# The role of programmed cell death in diabetes mellitus-induced erectile dysfunction: from mechanisms to targeted therapy

**DOI:** 10.1186/s12958-025-01368-1

**Published:** 2025-03-03

**Authors:** Jun Zhang, Sheng Xin, Jiaquan Mao, Xiaming Liu, Tao Wang, Jihong Liu, Xiaodong Song, Wen Song

**Affiliations:** 1 Department of Urology, Tongji Hospital, Tongji Medical College, Huazhong University of Science and Technology, Wuhan, Hubei China; 2 Institute of Urology, Tongji Hospital, Tongji Medical College, Huazhong University of Science and Technology, Wuhan, Hubei China

**Keywords:** Diabetes mellitus-induced ED, Apoptosis, Autophagy, Ferroptosis, Pyroptosis

## Abstract

Diabetes mellitus (DM) is a chronic metabolic disease that often leads to vascular endothelial injury and peripheral neuropathy. Erectile dysfunction (ED), a common condition in andrology, is frequently associated with DM. The incidence of diabetes mellitus-induced ED (DMED) is second only to the cardiovascular complications of diabetes. Compared to other types of ED, DMED presents with more severe symptoms, rapid progression, and notable resistance to phosphodiesterase type 5 inhibitors (PDE5is). Various forms of programmed cell death (PCD)—including apoptosis, autophagy, pyroptosis, and ferroptosis—play pivotal roles in the pathogenesis of DMED. An exacerbation of DMED is linked to critical irritants like advanced glycation end-products (AGEs) and reactive oxygen species (ROS) in the corpus cavernosum tissue. These irritants can spark anomalous activations of diverse PCDs, which damage primary corpus cavernosum cells like cavernous nerve cells, endothelial cells, and myocytes, leading to ED. Hence, we reviewed current knowledge on the mechanisms and therapeutic potential of targeting PCDs in DMED, aiming to advance strategies for enhancing erectile function.

## Introduction

Erectile dysfunction (ED), the persistent inability to achieve or maintain an erection sufficient for satisfactory sexual performance, affects millions of men worldwide [1, 2]. Diabetes is a chronic metabolic disease characterized by high blood sugar, accompanied by cardiovascular complications and non-vascular complications, including coronary heart disease, cardiomyopathy, kidney disease, and peripheral neuropathy [3]. Currently, approximately 463 million people globally are living with DM, a number projected to rise to 578 million by 2030 [4]. ED is a complication in male diabetes patients with an incidence second only to cardiovascular diseases. Diabetes mellitus-induced erectile dysfunction (DMED) occurs at a rate nearly three times higher in diabetic men than in non-diabetic counterparts and presents with more severe symptoms and faster progression [5, 6]. Currently, phosphodiesterase type 5 inhibitors (PDE5is) are the most commonly chosen drugs for treating ED, which work by inhibiting the degradation of cyclic guanosine monophosphate (cGMP) in the corpus cavernosum, thus amplifying the effect of cGMP in relaxing smooth muscles and prolonging erection time. They have shown evident efficacy in most ED patients. However, PDE5is have poorer efficacy in DMED patients, with about 30% of patients not responding to these drugs [7]. Therefore, this underscores an urgent need for alternative therapeutic strategies and a deeper understanding of DMED’s pathophysiology. Erection of the penis involves a complex neurovascular mechanism coordinated by nerves, vascular endothelium, and smooth muscles in a sophisticated physiological process. Sexual stimulation leads to the release of nitric oxide (NO) from neuronal nitric oxide synthase (nNOS) and endothelial nitric oxide synthase (eNOS). NO diffuses into adjacent smooth muscle cells, increasing cGMP concentration by activating guanylate cyclase. This activates a multitude of signaling cascades, reducing intracellular Ca^2+^ uptake and causing smooth muscle relaxation, leading to dilation of cavernosal sinuses and penile arteries. This also enhances positive feedback to promote eNOS synthesis, maintaining the eNOS/NO/cGMP pathway activity [8–10]. Consequently, any factors that can influence these above physiological processes might trigger ED.

Diabetes induces a spectrum of multifaceted and intricate pathological alterations within the penile corpora cavernosa, encompassing cavernosal nerve damage, endothelial cell dysfunction, impaired smooth muscle relaxation, and corpora cavernosa fibrosis [6, 11, 12]. One pivotal feature of tissues impacted by diabetes is the activation of oxidative stress alongside elevated concentrations of advanced glycation end-products (AGEs) and reactive oxygen species (ROS). These entities markedly impair the synthesis and bioavailability of eNOS-eNO, consequently undermining endothelium-dependent vasodilation mechanisms, which underscores a key facet of the pathophysiological underpinning of diabetes-associated ED [13, 14]. Moreover, under oxidative stress, endothelial and smooth muscle cells encounter considerable challenges, both in terms of cell quantity and quality. This adversarial interaction precipitates a decline in erectile function [11, 15].

Programmed cell death (PCD), which encompasses apoptosis, autophagy, pyroptosis, and ferroptosis (Table 1), helps maintain cellular homeostasis by ensuring the elimination of superfluous or damaged cells. Simultaneously, under stressful conditions, the activation of abnormal cell death can precipitate organ dysfunction, underscoring its dual impact [16, 17]. Hyperglycemia-induced overactivation of AGEs and ROS triggers PCDs in key cell types involved in erection, such as endothelial cells, smooth muscle cells, and neurons, contributing to the pathogenesis of DMED [18, 19]. Given this backdrop, exploring the molecular mechanisms of various PCDs within the penile corpora cavernosa tissue in hyperglycemic conditions and ameliorating erectile function through targeted regulation of PCDs hold substantial clinical significance. From the standpoint of targeting PCDs, this review encapsulates diverse treatment approaches and their mechanisms in DMED.

**Table 1:** Main characteristics, morphological features, regulatory pathways, and biochemical changes of different kinds of cell deaths

Type of cell deaths	Apoptosis	Autophagy	Ferroptosis	Pyroptosis
**Characteristics**	A classic form of programmed cell death serves to maintain homeostatic balance by regulating the cell life cycle under genetic control	Cellular degradation and recycling via engulfment by autophagosomes and digestion by lysosomes	An iron-mediated and excessive intracellular oxidation-associated programmed cell death	Characterized by the involvement of Gasdermin family proteins and inflammatory reaction.
**Morphological features**				
Cell membrane	Membrane blebbing	Normal membrane structure	Cell rounding, pore formation	Cell swelling, pore formation, rupture of plasma membranes
Cytoplasm	Cell shrinkage, formation of apoptotic bodies, mitochondria damage	Autophagic vacuole with double membranes	Cell swelling, mitochondria damage	Release of intracellular contents, mitochondria damage
Nucleus	Nuclear condensation, DNA fragmentation, chromatin condensation	Remains intact	Remains intact	DNA fragmentation, chromatin condensation
Mitochondria	MOMP	Mitochondria can be degraded by autolysosomes	Mitochondrial atrophy, reduced mitochondria crista, improved mitochondrial membrane density	Mitochondrial dysfunction due to ROS and inflammatory factors
**Main pathways**	Mainly involving the mitochondria-mediated pathway and death receptor-mediated pathway	Primarily involving macroautophagy, microautophagy, and chaperone-mediated autophagy	Primarily involving the GSH/GPX4 pathway, the NADH/FSP1/CoQ10 pathway, iron metabolism regulation, fatty acid oxidation pathway	NLRP3-associated classic inflammasome and non-classic inflammasome
**Triggers**	Intrinsic lethal stimuli (oxidative stress, metabolic stress, and DNA damage) or activation of death receptors	ULK1 complex activation	Inhibition of GPX4, inhibition system Xc-, excessive ferritinophagy, overoxidation of PUFAs	Formation of inflammasomes
**Key regulators**	Apoptosome, caspase-8, Bcl2 family proteins	ATG5,ATG7, VPS34, Beclin1	GPX4, system Xc-, FSP1, TFRC, NCOA4, ACSL4, LPCAT3	Caspase-1, caspase-4/5/11, gasdermin family protein, and active IL-1β/IL‐18
**Biochemical features**	Caspase activation, DNA fragment, mitochondrial membrane potential alterations, MOMP, release of cytochrome c	LC3-I lipidation, formation of autolysosomes, improved lysosomal activity	GSH deficiency, iron overload, accumulation of ROS, and lipid peroxidation	Caspase-dependent, gasdermin cleavage, formation of inflammasome, release of IL-18 and IL-1β
**Inflammatory nature**	Non-inflammatory	Non-inflammatory	Non-inflammatory	Highly inflammatory

## Apoptosis

### Overview of apoptosis

As early as 1972, Kerr and colleagues introduced the concept of apoptosis to describe a form of cell death that is morphologically distinct. Apoptosis is an evolutionarily conserved PCD [20]. Characterized by cell shrinkage, chromatin condensation, DNA fragmentation, and the formation of apoptotic bodies, apoptosis plays a crucial role in maintaining cellular homeostasis [21, 22]. The underlying biological processes of apoptosis are intricate, involving a complex orchestration of interactions among various proteins, signaling molecules, and cascade reactions of signaling pathways. Presently recognized, the apoptotic pathways in eukaryotic cells mainly involve two routes: the intrinsic pathway and the extrinsic one.

### Regulatory mechanisms of apoptosis

The intrinsic pathway is triggered by intracellular stress signals that affect mitochondrial outer membrane permeability. This process is regulated by the B-cell lymphoma-2 (Bcl-2) protein family, which includes pro-apoptotic members like Bcl-2 associated X protein (Bax) and Bcl-2 antagonist/killer (Bak), and anti-apoptotic members like Bcl-2 and B-cell lymphoma-extra large (Bcl-xL) [23–25]. Moreover, BH3-interacting domain death agonist (Bid) and Bcl-2-like 11 (Bim) are constituents of the B-cell lymphoma-2 homology domain 3 (BH3)-only proteins. Their mandate involves the nuanced regulation of pro-apoptotic and anti-apoptotic proteins. The interaction of these proteins determines the direction of the apoptotic process and profoundly influences the destiny of the cell [26]. A pivotal turn occurs when external apoptotic signals impinge upon the mitochondria. Under this influence, Bcl-2 protein is rendered inactive; simultaneously, pro-apoptotic proteins trigger mitochondrial outer membrane permeabilization (MOMP) through engendering a series of events, including membrane potential alterations, mitochondrial permeability transition pore (PTP) opening. These progressions culminate in the consequent release of cyt-c [27], which then combines with pro-caspase-9 and apoptotic protease activating factor-1 (Apaf-1) to form the apoptosome, unleashing a cascade activation of cysteine proteases [28]. The consequential activation of cleaved-caspase-9 facilitates the cleavage of pro-caspase-3/6/7, inducing cell apoptosis [29].

**Fig. 1:**
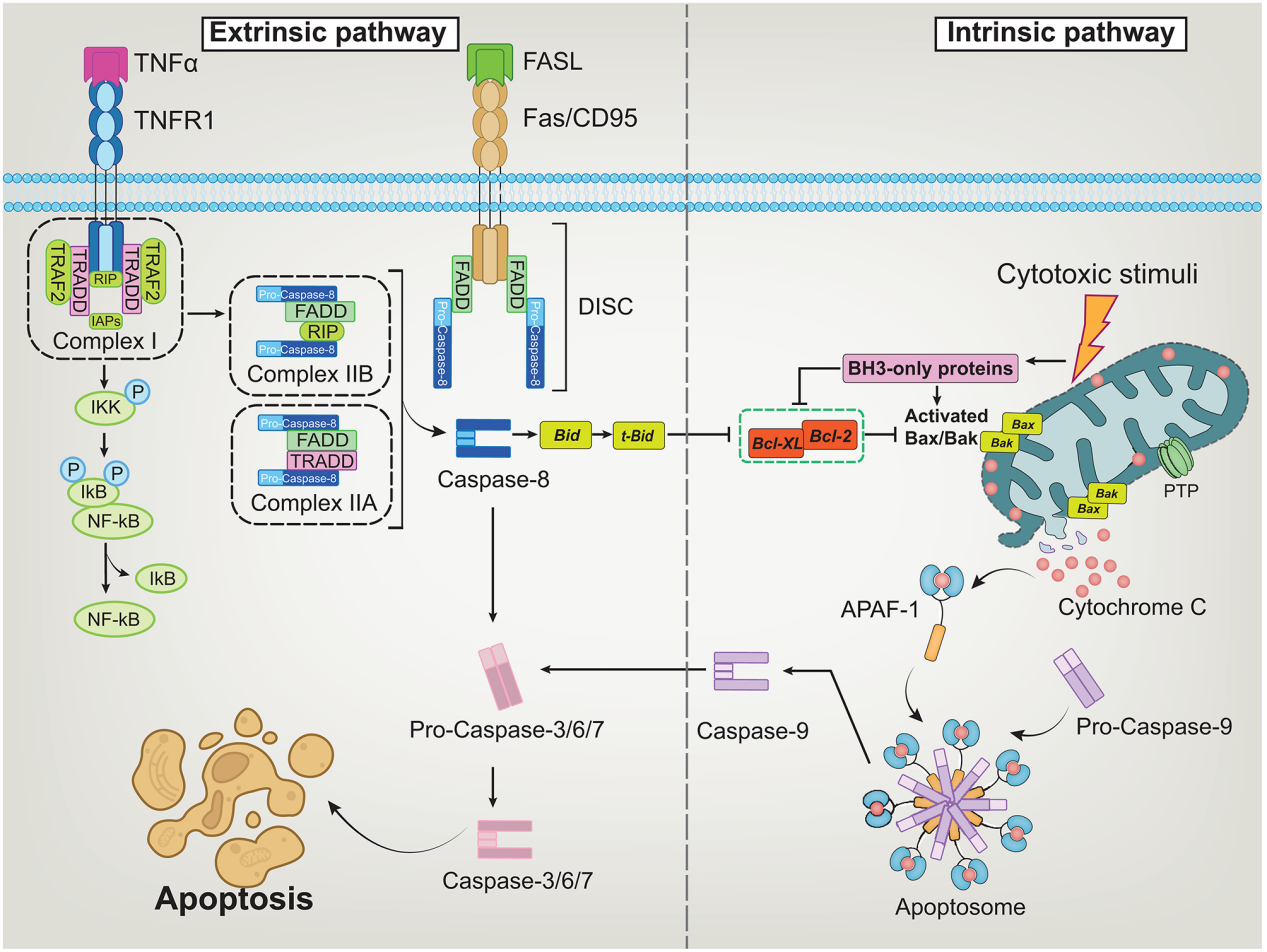
Molecular mechanisms of intrinsic and extrinsic pathways in cellular apoptosis. A. The extrinsic pathway is triggered when death receptors (e.g., Fas, TNFR1) and death ligands (e.g., FasL, TNFα) bind together. This interaction leads to the formation of DISC, complex I, and complex IIA sequentially. Subsequently, the activated caspase-8 facilitates the cleavage of pro-caspase-3/6/7, resulting in cell death. Alternatively, complex IIA also serves to activate caspase-8, which cleaves Bid into tBid, setting off the mitochondrial pathway and thereby orchestrating an indirect induction of cell death; B. The intrinsic pathway is triggered by intracellular stress signals that affect the balance of Bcl-2 family proteins. Pro-apoptotic members Bax and Bak promote MOMP, while anti-apoptotic proteins Bcl-2 and Bcl-xL inhibit this process. BH3-only proteins modulate this balance by inhibiting anti-apoptotic proteins and activating pro-apoptotic ones. When pro-apoptotic signals predominate, MOMP occurs, leading to the release of cytochrome c into the cytosol. Cytochrome c combines with APAF-1, facilitating the formation of the oligomer. Pro-caspase-9 is then recruited to form the apoptosome, and its cleavage converts into initiator caspase-9, which then activates effector caspase-3/6/7, executing the apoptotic program

The extrinsic pathway unfolds through the interaction between death receptors and their ligands. Death receptors belong to the tumor necrosis factor receptor (TNFR) superfamily, mainly including fatty acid synthetase receptor (FasR) and TNFR [30]. Fatty acid synthetase ligand (FasL) binds to the FasR (also known as CD95) to recruit the Fas-associated death domain protein (FADD) and recruits downstream interactor pro-caspase-8 to form the death inducing signaling complex (DISC) through the homotypic interactions of the death effector domain (DED). Once pro-caspase-8 is auto-cleaved, it produces activated cleaved-caspase-8, initiating the executive stage of apoptosis [31]. In the TNF-mediated pathway, after the TNFR interacts with its ligand, the TNFR1-associated death domain protein (TRADD) recruits FADD, initiating the cascade activation of cysteine proteases and leading to cell apoptosis [32]. TRADD orchestrates the generation of complex I by facilitating the binding of cellular inhibitors of apoptosis proteins (IAPs), receptor-interacting protein kinase (RIP1), and TNF receptor-associated factors (TRAFs), which activates the nuclear factor kappa-B (NF-κB) pathway[33]. Complex I can assemble into two different cytoplasmic apoptotic assemblies, complex IIA, and complex IIB, which trigger the apoptosis process by initiator caspase-8 or pro-apoptotic protein Bid [34] (Fig. 1).

### Apoptosis and ED

Dysregulated apoptosis is implicated in the pathogenesis of various diseases, including DMED. Excessive apoptosis in penile tissue significantly contributes to DMED progression, and interventions targeting apoptotic pathways have shown potential in improving erectile function (Table 2). Li H and others found that diabetic mice with ED exhibited significantly higher expression of caspase-3 and its active form, cleaved caspase-3, in the corpus cavernosum compared to controls [35]. Additionally, it was found that the expression of the anti-apoptotic protein Bcl-2 greatly decreased in DMED rats, whilst the levels of the pro-apoptotic proteins Bax and caspase-3 increased. However, these parameters were reversed following treatment with leech and centipede granules (LCG) [36]. Further research revealed that smooth muscle cells of the penile cavernous body in DMED rats showed a significantly increased apoptosis rate in flow cytometry detection. The immunofluorescence intensity of caspase-3 also increased, and berberine improved the erectile function of DMED rats by inhibiting cell apoptosis in the penile cavernous body [37]. Recent studies have shown that nitro-oleic acid, salidroside, and MSCT combined with probucol ameliorated DMED by upregulating the nuclear factor erythroid 2-related factor 2 (Nrf2) pathway and inhibiting apoptosis [38–40]. Relaxin-2, isorhamnetin, and Gynochthodes officinalis (F.C.How) Razafim. & B. Bremer (G. officinalis) alleviated DMED by targeting endothelial dysfunction (via phosphatidylinositol 3-kinase (PI3K)/protein kinase B (AKT)/eNOS pathway) and apoptosis (via Bcl-2/Bax regulation and ROS reduction) [41–43]. Endothelial cell apoptosis was also overactivated in DMED patients, evidenced by elevated levels of endothelial microparticles (EMPs) in their plasma, which were specific markers of endothelial cell apoptosis [44].

**Table 2:** Drugs or treatment procedures of DMED related to the regulation of PCDs

Drugs/ compound/ procedure	Target pathway	In vivo/ In vitro	Effects on apoptosis	Treatment effects	Ref.
Berberine	Downregulating the SPHK1/S1P/S1PR2 pathway, the MAPK pathway	In vivo/ In vitro	Downregulate apoptosis	Improved endothelial function, inhibited the expression of TGFβ1, collagen I/IV, and then ameliorated ED	[37]
LCG	Downregulating the CaSR/PLC/PKC pathway	In vivo	Downregulate apoptosis	Increased endothelial function, alleviated vascular and tissue fibrosis of the penis	[36]
HUCB ECFCs	**-**	In vivo/ In vitro	Downregulate apoptosis	Enhanced proliferation of endothelial and smooth muscle cells and partially recovered the functionality of nerve cells	[45]
JTE-013	Downregulating the RhoA/ ROCK/p-MYPT1 pathway	In vivo	Downregulate apoptosis	Inhibited smooth muscle contraction and severe corporal fibrosis, decreased the expression of S1PR2, improved erectile function	[46]
MDL-28,170	**-**	In vivo	Downregulate apoptosis	Attenuated calpain activity, reversed the damage to endothelial and smooth muscle cells, and improved erectile function	[47]
Rapamycin	-	In vivo	Downregulate apoptosis	Reduced the content of pro-apoptotic proteins and inhibition of apoptotic proteins showed opposite results	[48]
Nitro-oleic acid	Upregulating the Nrf2 pathway, the NO/cGMP pathway	In vivo	Downregulate apoptosis	Elevated eNOS phosphorylation levels and enhanced NO production, with anti-oxidative stress effect	[38]
Salidroside	Upregulating the Nrf2 pathway	In vivo/ In vitro	Downregulate apoptosis	Anti-oxidative stress effect	[39]
MSCT + probucol	Upregulating the Nrf2 pathway	In vivo	Downregulate apoptosis	Resulted in a higher level of Bcl-2 and a lower of Bax, and reduced downstream expression of caspase-3 and cleavedcaspase-3	[40]
Relaxin-2	Upregulating the PI3K/AKT/eNOS pathway, downregulating the RhoA/ ROCK pathway	In vivo/ In vitro	Downregulate apoptosis	Restored endothelial cells and smooth muscle cells	[41]
Isorhamnetin	Upregulating the PI3K/AKT/eNOS pathway	In vivo/ In vitro	Downregulate apoptosis	Restored endothelial cells and smooth muscle cells, with anti-oxidative stress effect	[42]
G. officinalis	Upregulating the PI3K/AKT/eNOS pathway	In vivo/ In vitro	Downregulate apoptosis	Restored endothelial cells and smooth muscle cells, with anti-oxidative stress effect	[43]
Methyl protodioscin	Downregulating the c-Myc/AKAP12 pathway	In vivo/ In vitro	Downregulate apoptosis	Elevated eNOS phosphorylation levels and enhanced NO production, restored vascular endothelial cells and vascular smooth muscle cells	[49]
USCs	**-**	In vivo/ In vitro	Facilitate autophagy	Reversed endothelial dysfunction and enhanced ED	[72]
MSCT + ESWT	Upregulating the PI3K/AKT/mTOR pathway	In vivo	Facilitate autophagy	Enhanced vascular generation and regeneration in the corpus cavernosum, accompanied by upregulation of VEGF	[50]
MSCT + probucol	Upregulating the Nrf2 pathway	In vivo	Facilitate autophagy	Enhanced the self-antioxidant ability of MSCs, improved the transplanted microenvironment of MSCs to prolong their survival period, and enhanced therapeutic effectiveness	[40]
Simvastatin	Upregulating the AMPK-SKP2-CARM1 pathway	In vivo/ In vitro	Facilitate autophagy	Lowered the extent of fibrosis in the corpus cavernosum, and enhanced erectile function	[51]
Rapamycin	Downregulating the AKT/mTOR pathway, the AMPK/mTOR pathway, and the mTOR/p70S6K pathway	In vivo	Facilitate autophagy	Promoted protective autophagy, downregulated the level of apoptosis, and enhanced erectile function	[48]
Liraglutide	Downregulating the RhoA/ROCK pathway	In vivo/ In vitro	Facilitate autophagy	Alleviated smooth muscle dysfunction, decreased ROS production and NOXs levels, irrespective of its glucose-lowering action	[52]
Si-hmgcs2	Downregulating the mTOR pathway	In vivo/ In vitro	Facilitate autophagy	Increased eNOS phosphorylation levels and enhanced NO production	[53]
MiRNA-100	Downregulating the mTOR pathway	In vivo/ In vitro	Facilitate autophagy	Anti-fibrotic, anti-inflammatory effects, and improvement of endothelial function	[54]
BaTCG nanosystem	Upregulating the Pink1/Parkin pathway	In vivo/ In vitro	Facilitate autophagy	Restored endothelial cells and smooth muscle cells, with anti-inflammatory and anti-oxidative stress effects	[55]
Icariside II	Upregulating the PI3K/AKT/mTOR pathway	In vivo	Downregulate autophagy	Alleviated oxidative stress by decreasing ROS, and increasing SOD, improved growth curve, delayed fibrosis, and downregulated Beclin-1 expression	[56]
Icariside II	Downregulating the mTOR pathway	In vivo	Downregulate autophagy	Promoted smooth cell growth, downregulated AGEs, and decreased the numbers of autophagosomes	[57]
GKT-137,831	Upregulating the PI3K/Akt/mTOR pathway	In vivo	Downregulate autophagy	Reversed excessive autophagy and inflammatory responses	[58]
Fer-1	**-**	In vivo/ In vitro	Downregulate ferroptosis	Promoted the proliferation of corpus cavernosum smooth muscle cells	[59]
GPX4 lentivirus	Downregulating the ACSL4-LPCAT3-LOX pathway, the RhoA/ ROCK pathway	In vivo	Downregulate ferroptosis	Reversed the damage to the number and function of endothelial cells and smooth muscle cells, and inhibited fibrosis in the corpus cavernosum	[60]
Hesperidin	Upregulating the Nrf2–HO-1/GPX4 pathway, downregulating the RhoA/ ROCK pathway	In vivo/ In vitro	Downregulate ferroptosis	Restored endothelial/smooth muscle cell function and attenuated oxidative stress and fibrosis	[61]
NEDD4	The NEDD4-GPX4 ubiquitination-proteasome pathway, downregulating the RhoA/ ROCK pathway	In vivo/ In vitro	Downregulate ferroptosis	Regulated NEDD4-mediated GPX4 ubiquitination to preserve CCSMC function	[62]
HUCMSCs	Upregulating the SLC7A11/GPX4/ ACSL4 pathway	In vivo/ In vitro	Downregulate ferroptosis	Promoted the proliferation of smooth muscle cells, reduced oxidative stress and fibrosis, normalized mitochondrial morphology	[63]
Yimusake	Downregulating the NLRP3/caspase-1/IL-1β pathway	In vivo	Downregulate pyroptosis	Reduced inflammatory cell death and restored endothelial and smooth muscle function	[64]

SPHK1, sphingosine kinase 1; S1P, sphingosine-1-phosphate; S1PR2, S1P receptor 2; MAPK, mitogen-activated protein kinases; TGFβ1, transforming growth factor-β1; CaSR, calcium-sensing receptor; PLC, phospholipase C; PKC, protein kinase C; HUCB ECFCs, human umbilical cord blood derived endothelial colony forming cells; p-MYPT1, phosphorylated forms of myosin phosphatase targeting subunit 1; VEGF, vascular endothelial-derived growth factor; p70S6K, 70-kDa ribosomal protein S6 kinase; SKP2, S-phase kinase-associated protein 2; CARM1, co-activator-associated arginine methyltransferase 1; SOD, superoxide dismutase; Fer-1, ferrostatin-1; Si-hmgcs2, small interfering RNA targeting 3-hydroxy-3-methylglutaryl-CoA synthase 2; Pink1, PTEN-induced putative kinase 1; AKAP12, A-kinase anchor protein 12

## Autophagy

### Overview of autophagy

Autophagy, also termed programmed cell death type II, is a vital intracellular degradation process essential for cellular homeostasis. This process involves the encapsulation and transportation of anomalous proteins and damaged organelles within cells to lysosomes for degradation and recycling [65]. These actions are crucial for both quality and quantity control of cellular components under physiological conditions. Under various stressful conditions, autophagy is activated to preserve cell metabolism and ensure normal survival by efficiently managing cellular waste and damage [66]. There are three forms of autophagy in cells: macroautophagy, microautophagy, and chaperone-mediated autophagy. The term “autophagy” typically refers to macroautophagy due to its significant role in cellular maintenance and survival.

### Regulatory mechanisms of autophagy

Autophagy mainly includes the following steps: induction of autophagy, assembly of autophagosomes, fusion of the autophagosome with the lysosomal membrane, and degradation and recirculation of autolysosome contents [67]. When intracellular and extracellular stimuli cause a response in the mammalian target of rapamycin (mTOR) and the 5’AMP-activated protein kinase (AMPK) signaling pathway, the autophagy-initiating UNC-51-like kinase (ULK1) complex gets activated. In this context, autophagy-related proteins 13 (ATG13) secures ULK1 to a pre-autophagosomal structure (PAS), which subsequently binds with multiple ATGs. Subsequently, the class III phosphatidylinositol 3-kinase (PI3K-III) complex is activated, promoting the formation of autophagosome. The extension of the autophagosome is mediated by the ATG12 conjugation system and microtubule-associated protein 1 light chain 3 (LC3) conjugation system, forming a double-membraned structure of the mature autophagosome.

**Fig. 2:**
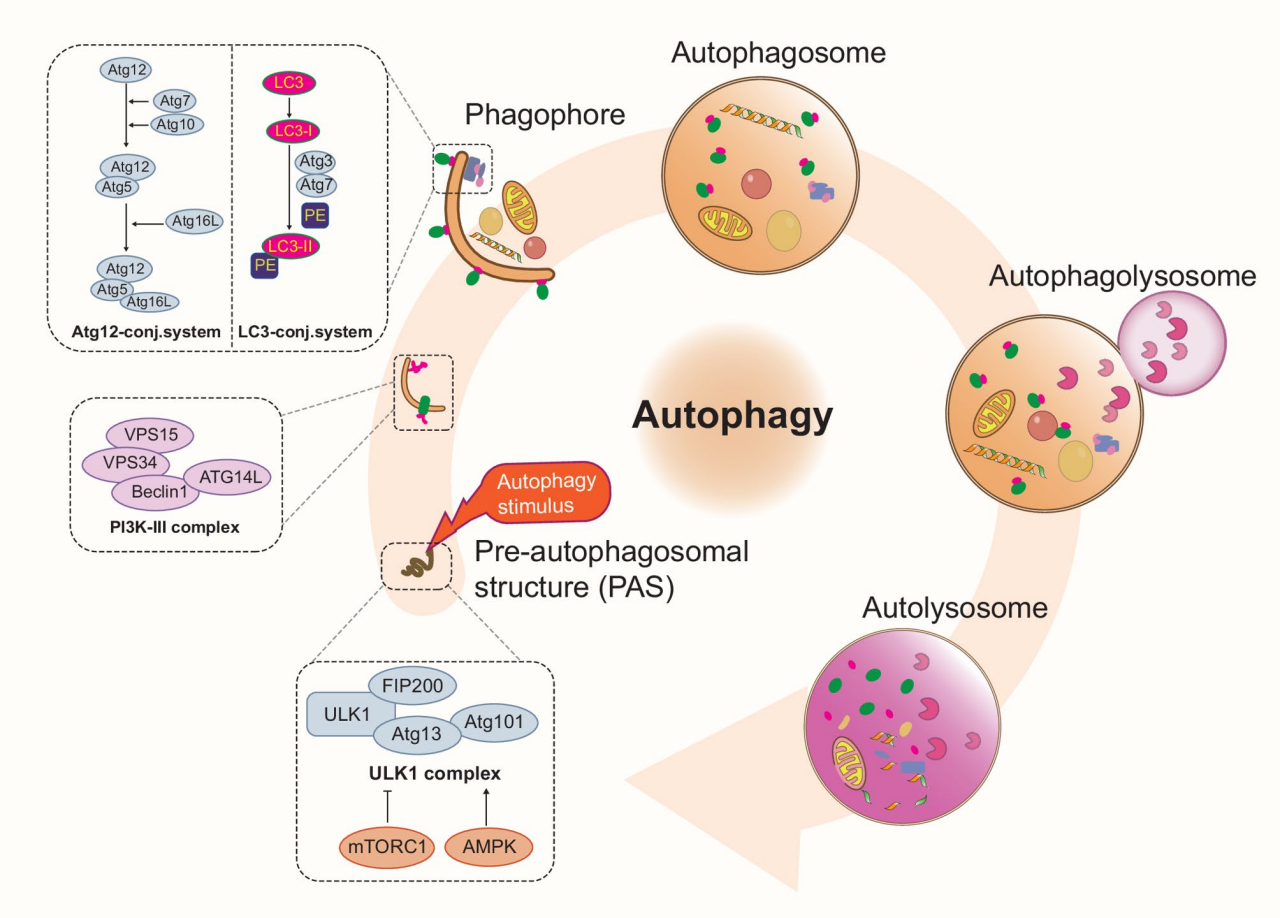
Molecular regulatory mechanisms of autophagy. Autophagy proceeds through several key stages: initiation and activation of autophagy, assembly, elongation, and maturation of autophagosomes, docking, and fusion with lysosomes, and finally, degradation and recycling of cargo within the autolysosome. Autophagy is vital for the renewal and rejuvenation of old and damaged cellular components, functioning as the cell’s quality control and recycling system

Autophagosomes need to bind to LC3, a hallmark of the cellular autophagy process, in order to engulf cytoplasmic materials. During autophagy formation, LC3-I is lipidated under the action of multiple ATGs, converting to LC3-II which binds to the autophagosome membrane [34, 68]. Mature autophagosomes then fuse with lysosomes to form autolysosomes, where the contents are degraded and recycled for cellular use [69] (Fig. 2).

### Autophagy in ED

Emerging research suggested that autophagy was implicated in the microvascular complications of diabetes [70, 71], yet its precise molecular mechanisms in DMED remained to be fully elucidated. The role of autophagy in DMED is both nuanced and intricate. On one hand, under physiological conditions, it maintains cellular homeostasis in the corpus cavernosum by degrading and recycling cellular waste. On the other hand, dysregulated autophagy—either excessive activation or inhibition—can be detrimental to cell survival (Table 2). Recent research has suggested that injecting urine-derived stem cells (USCs) into the corpus cavernosum could upregulate the autophagy levels of endothelial cells, ameliorating endothelial dysfunction and improving erectile function [72]. Combined treatment with energy shock wave therapy (ESWT) and mesenchymal stem cell therapy (MSCT) has been reported to reverse ED in DMED rats by stimulating autophagy and suppressing apoptosis in cavernosal tissue [50]. Additionally, after injecting mesenchymal stem cells (MSCs) into the cavernous body and administering probucol, enhanced protective autophagy activity and reduced apoptosis of MSCs were observed [40]. Rapamycin, simvastatin, liraglutide, small interfering RNA targeting 3-hydroxy-3-methylglutaryl-CoA synthase 2 (Si-hmgcs2), and microRNA-100 (MiRNA-100) have been demonstrated to promote protective autophagy, improve cellular resistance to oxidative stress, and alleviate cavernous fibrosis, ultimately restoring endothelial function in DMED rats [48, 51–54]. Shi’s team developed the piezoelectric barium titanate (BaTiO3) and glucagon-like peptide-1 receptor agonists (GLP-1RAs) (BaTCG) nanosystem, which selectively eliminates damaged mitochondria (mitophagy) through its piezoelectric and mitochondrial targeting properties [55]. This strategy overcame the limitations of traditional antioxidant treatments that “only clear ROS without addressing the source for repair,” providing a promising integrated nanointervention for DMED.

However, recent academic research indicated that diabetes associated with hypertension damaged erectile function due to a confluence of factors, including upregulated levels of apoptosis and autophagy, oxidative stress, and inhibition of endothelial cell activity [18]. Zhang et al. found that icariin II alleviated CCSMC damage and improved erectile function by downregulating excessive mitochondrial autophagy and apoptosis in type 2 DMED rats. The possible mechanism: Autophagy and apoptosis partially overlap at the signaling level. For instance, the Bcl-2/Beclin-1 complex regulates both the initiation of autophagy and is closely related to mitochondrial outer membrane permeability and apoptosis initiation. In a high-glucose environment, excessive mTOR inhibition and significant upregulation of Beclin-1 trigger excessive autophagy and increase the likelihood of CCSMC apoptosis [56, 57, 73]. In addition, inhibition of NADPH oxidase 1/4 (NOX1/4) attenuates ED caused by ROS overproduction by downregulating excessive autophagy and apoptosis and enhancing eNOS expression in endothelial cells [58]. In summary, autophagy has a positive role in clearing damage and maintaining homeostasis, but it may also induce excessive apoptosis under pathological conditions. Future research on DMED treatment will need to strike a delicate balance between inhibiting “harmful excessive autophagy” and preserving “normal, moderate autophagy”.

## Ferroptosis

### Overview of ferroptosis

Ferroptosis is a form of programmed cell death characterized by iron-dependent lipid peroxidation, first identified by Dixon et al. in 2012. Unlike apoptosis, autophagy, or necrosis, ferroptosis cannot be prevented by inhibitors of these pathways but can be reversed by iron chelators such as deferoxamine [74, 75]. Morphologically, it is marked by mitochondrial shrinkage, reduced cristae, and increased membrane density [76, 77]. The process arises from antioxidant deficiencies and excessive intracellular oxidation, leading to the accumulation of lipid peroxides (LPO) due to Fe²⁺-catalyzed reactions. Excessive LPO causes oxidative damage and loss of cell membrane integrity [75].

### Regulatory mechanisms of ferroptosis

Recent studies indicated that imbalances in iron homeostasis, inhibition of amino acid transport, excessive oxidation of polyunsaturated fatty acids (PUFA) in lipids, and glutathione (GSH) metabolic dysregulation were drivers of ferroptosis [78, 79]. The extensive ROS production ultimately cause ferroptosis in cells. Overoxidation of PUFAs, accumulation of free iron, and consumption of GSH are considered central to ferroptosis [80].

The GSH/glutathione peroxidase 4 (GPX4) axis plays a crucial role in maintaining phospholipid redox homeostasis [81]. The solute carrier proteins solute carrier family 3 member 2 (SLC3A2) and solute carrier family 7a member 11 (SLC7A11) form the glutamate-cysteine transporter (System Xc-), assisting cysteine’s entry into cells. Once inside the cell, cysteine is catalyzed into GSH through the synergistic activity of glutamate-cysteine ligase (GCL) and glutathione synthetase (GSS) [82, 83]. GSH then functions as a reductive helper for glutathione peroxidase 4 (GPX4), enabling it to eliminate lipid peroxides effectively. A disruption in this process, especially when System Xc- is suppressed, can lead to inadequate GSH generation. This deficiency, in turn, results in ROS accumulation, ultimately triggering ferroptosis [84, 85].

Iron metabolism is another critical factor. Throughout the entire process of iron absorption, storage, and excretion, each step can influence ferroptosis [86, 87]. Fe^3+^ initially binds tightly with transferrin (Tf), then is transported to the endosome mediated by the transferrin receptor 1 (TfR1), where Fe^3+^ is catalytically converted to its reduced form, Fe^2+^. Subsequently, Fe²⁺ is released into the cytoplasm through divalent metal transporter 1 (DMT1) for utilization, storage in ferritin, or export via ferroportin 1 (FPN1) [88, 89]. Upon the activation of transferrin, an abundance of iron ions flows into the cell via the transferrin receptor, or certain molecular mechanisms that promote the release of stored iron, ultimately resulting in an overload of intracellular iron ions, leading to ferroptosis [90]. For instance, nuclear receptor coactivator 4 (NCOA4), upon binding with ferritin, directs it to the autophagolysosome. A process known as ferritinophagy releases the iron contained in ferritin as free iron; aberrant ferritinophagy activation can result in an excessive buildup of free iron in the cytoplasm, increasing the susceptibility of cells to ferroptosis [91, 92].

Overoxidation of polyunsaturated fatty acids (PUFAs) also drives ferroptosis, especially arachidonic acid (AA) [93]. Under the catalytic action of acyl-CoA synthetase long-chain family member 4 (ACSL4), lysophosphatidylcholine acyltransferase 3 (LPCAT3), and lipoxygenase (LOX), LPO is formed [94, 95]. During the enzyme-mediated lipid peroxidation process, Fe²⁺ facilitates the generation of alkoxyl radicals from lipid peroxides, initiating a chain reaction of lipid peroxidation that culminates in ferroptosis [85].

**Fig. 3:**
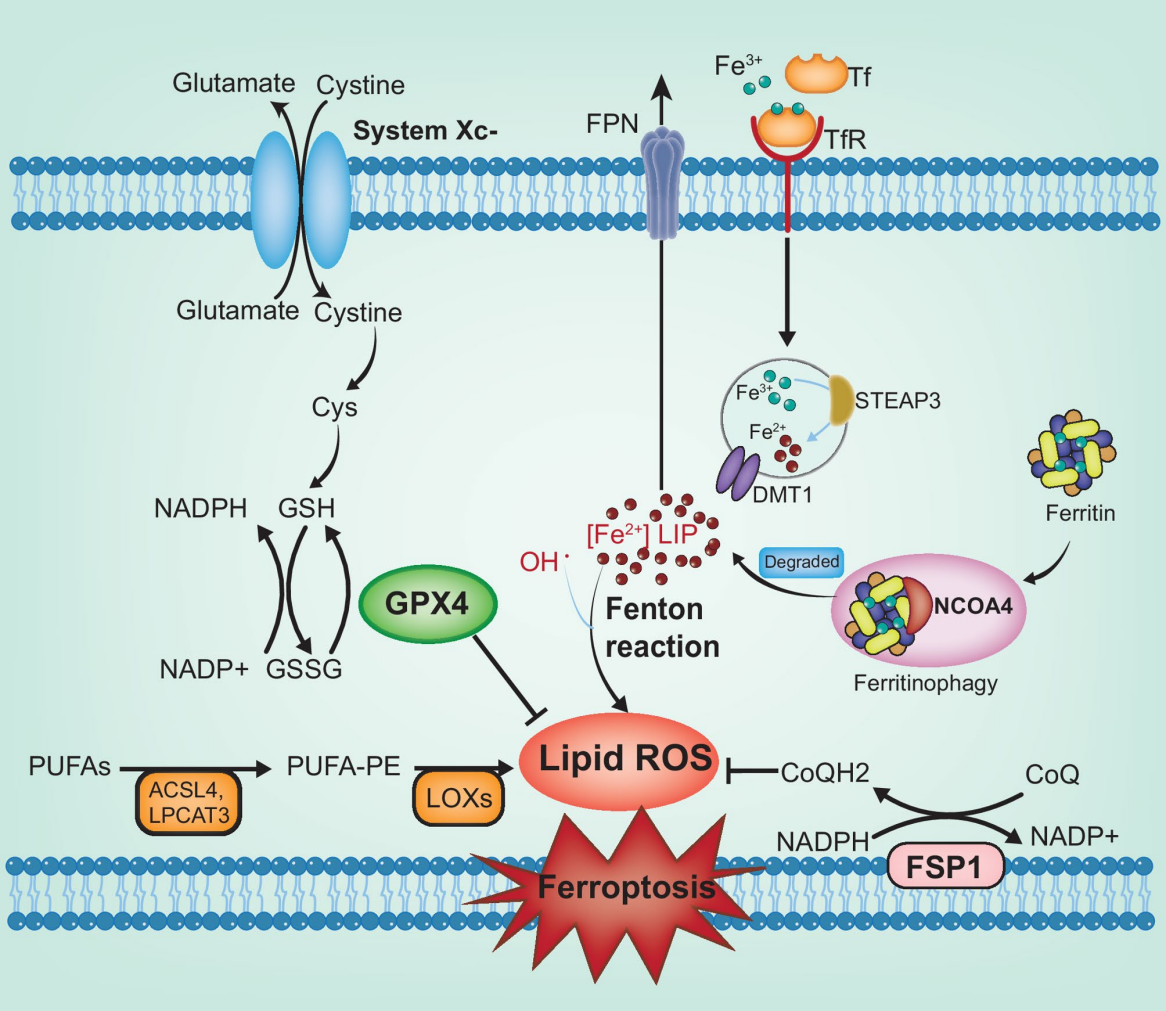
Molecular mechanisms regulating ferroptosis. Ferroptosis is primarily modulated through pathways related to glutathione consumption, free iron accumulation, or lipid peroxidation. The GSH/GPX4 axis is crucial: inhibition of system Xc- reduces intracellular cystine levels, leading to decreased GSH synthesis and disruption of GPX4’s antioxidant activity. Iron metabolism also plays a pivotal role. NCOA4-mediated ferritinophagy and imbalances in iron uptake and export result in excess free iron. Once iron levels surpass the buffering ability of ferritin, excess iron facilitates lipid peroxide generation via the Fenton reaction. In fatty acid oxidation pathways, enzymes such as ACSL4 and LPCAT3 catalyze PUFAs into PUFA-PE, which are subsequently oxidized by LOXs into LPO. The NADH/FSP1/CoQ10 axis counteracts ferroptosis by reducing CoQ10 to its active form, CoQ10H₂, capturing lipid peroxyl radicals and preventing lipid peroxidation

In addition, recent research has revealed that the NADH/FSP1/CoQ10 pathway functions independently from the GSH/GPX4 pathway [96]. Positioned on the plasma membrane, ferroptosis suppressor protein 1 (FSP1) catalyzes the production of the hydroquinone form of CoQ (CoQH2) through the reductase nicotinamide adenine dinucleotide (NADPH), a lipophilic radical-trapping antioxidant, to counteract ferroptosis [97, 98] (Fig. 3).

### Ferroptosis in ED

Ferroptosis has been confirmed to cause structural and functional damage in various disease models, particularly in complications related to diabetes [91]. Studies have shown that the administration of sulforaphane could inhibit cardiomyocyte ferroptosis to prevent diabetic cardiomyopathy [99]. Moreover, tissue alterations and organ dysfunction in diabetic nephropathy and diabetic osteoporosis are also closely related to ferroptosis [100, 101].

Xu et al. performed RNA sequencing and differential gene expression analysis on the corpus cavernosum of DMED rats, revealing significant enrichment of ferroptosis-related genes. Further in vitro validation showed that after applying ferroptosis inhibitors to cavenosum smooth muscle cells (CCSMCs), there was an upregulation of GPX4 and a significant reduction in the levels of ferroptosis-related proteins [59]. This evidence suggested that there may be a subtle link between DMED and ferroptosis. Subsequent studies found that in DMED rat models, the levels of iron ions and lipid peroxidation in tissues were significantly higher than those in normal rats. The application of GPX4 lentiviral vectors (GPX4-LV) was found to be effective in promoting the proliferation of smooth muscle and endothelial cells, improving penile tissue fibrosis, reversing oxidative stress, and effectively ameliorating erectile function in DMED rats [60]. Xin et al. found that hesperidin alleviates DMED by activating the Nrf2-heme oxygenase-1 (HO-1)/GPX4 axis to suppress oxidative stress and ferroptosis, thereby restoring endothelial/smooth muscle cell function and reducing cavernous fibrosis [61]. Xu et al. identified neural precursor cell-expressed developmentally downregulated protein 4 (NEDD4)-mediated GPX4 ubiquitination as a central driver of HG-induced ferroptosis in CCSMCs, exacerbating DMED through oxidative stress, Ras homolog gene family member A (RhoA)/Rho-associated coiled-coil-containing kinase (ROCK) activation, and fibrosis [62]. These results underscored GPX4 as a promising target for regulating ferroptosis in the treatment of DMED. Moreover, studies have shown that human umbilical cord mesenchymal stem cells (HUCMSCs) treatment effectively reversed the overexpression of genes related to ferroptosis and the downregulation of the ferroptosis suppressor gene. Additionally, this treatment improved fibrosis and oxidative stress in corpus cavernosum tissues and enhanced the relaxation function of the smooth muscle in both type 1 and type 2 diabetic rats with ED [63]. This suggested that stem cell therapy could emerge as a potent and efficient new treatment option for DMED patients in the future (Table 2).

## Pyroptosis

### Overview of pyroptosis

In 2001, Cookson showed that the form of cell death caused by salmonella typhimurium infection in macrophages was dependent on caspase-1 activity and formally defined it as “pyroptosis“ [102]. Its characteristics include cell swelling and the release of cytoplasmic content and inflammatory factors, initiating an inflammatory cascade that leads to cell damage [103, 104]. Additionally, Shao and colleagues discovered that in the cytoplasm, caspase-4/5/11 was activated by lipopolysaccharide (LPS), and induced pyroptosis by cleaving the Gasdermin protein family. They ultimately defined pyroptosis as programmed cell necrosis executed by the GSDM family [105, 106]. GSDMs have an N-terminal pore-forming domain (GSDMs-NT) and a C-terminal inhibitory domain (GSDMs-CT). Upon cleavage by cysteine protease, the active GSDMs-NT is produced, which disrupts the phospholipid bilayer, resulting in cell lysis and the release of inflammatory mediators [107, 108]. With deeper research into pyroptosis, it’s found that the mechanism of pyroptosis includes the canonical pathway and the non-canonical pathway.

### Regulatory mechanisms of pyroptosis

The canonical pathway, a caspase-1-mediated cell death, is initiated by danger signals stimulating the pattern recognition receptors (PRRs), which then assemble with the apoptosis-related speck-like protein (ASC) and pro-caspase-1 to form inflammasomes. AGEs and ROS produced in a high glucose environment can activate the assembly of NOD-like receptor NLR family pyrin domain-containing 3 (NLRP3) inflammasomes [109, 110]. ASCs, primarily composed of the pyrin domain (PYD), caspase activation, and recruitment domain (CARD), serve as bridging proteins that represent the activation of inflammasomes [111]. Once PRR is activated, it interacts with ASC, recruits and activates procaspase-1 to cleave and produce the active caspase-1, which then catalyzes the generation of the key pyroptosis-inducing protein GSDMD-NT and active inflammatory factors, leading to cell membrane perforation, cytoplasmic content leakage, and triggering a cascade of inflammatory responses [112, 113].

**Fig. 4:**
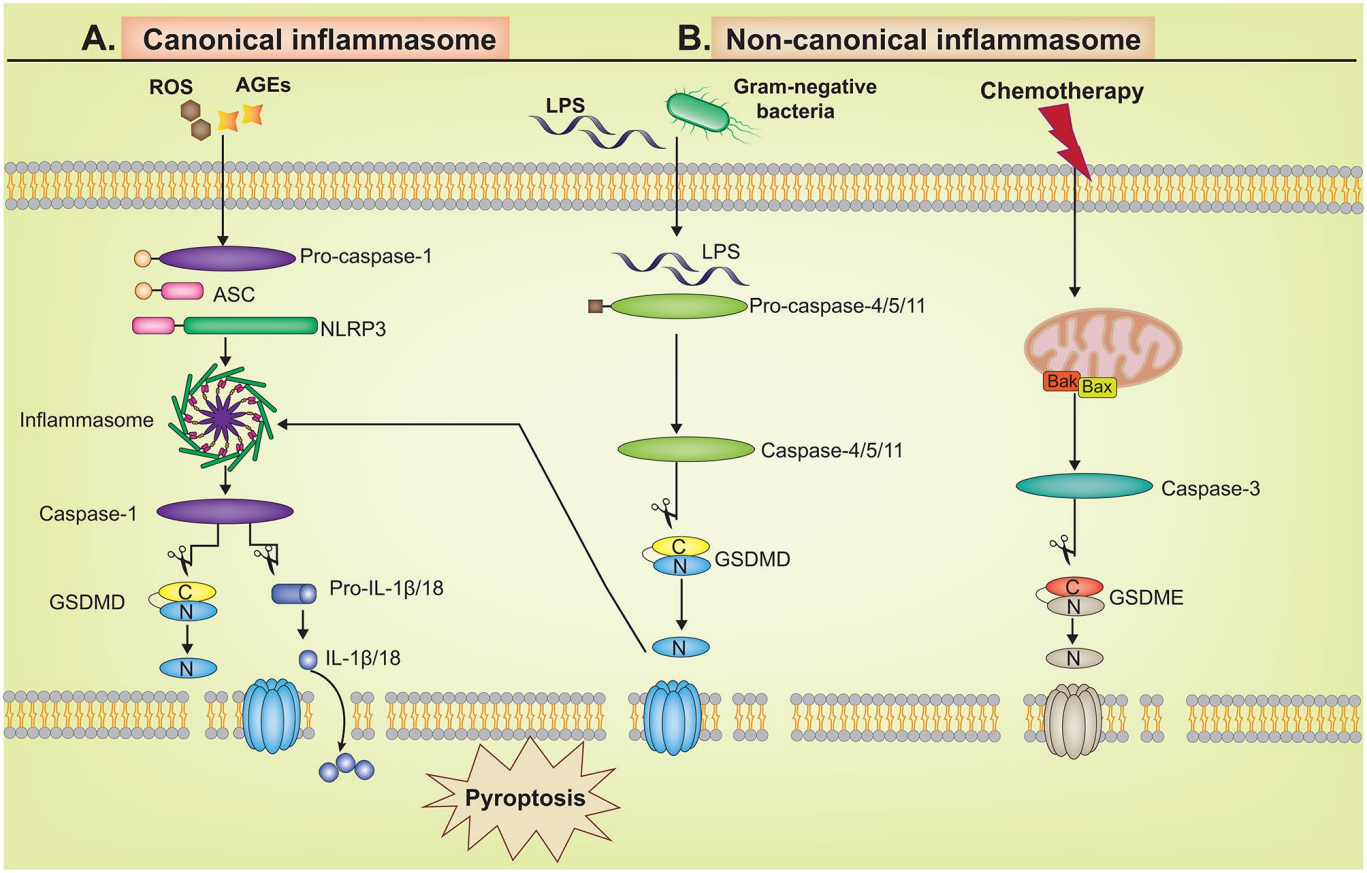
Two different molecular mechanisms of pyroptosis. The canonical and non-canonical pathways. **A**. In the canonical pathway, NLRP3 responds to external stimulation signals and then recruits pro-caspase-1 through bridging protein ASC. Activated caspase-1 directly cleaves GSDMD to produce GSDMD-NT, which can drill into the cell membrane and form membrane pores. Caspase-1 also processes pro-IL-1β/18 into their mature forms, which are released through the above pore structure, leading to pyroptosis and amplifying the inflammatory response; **B**. In the non-canonical pathway, caspase-4/5/11 is activated by interacting with LPS in the cytoplasm, and cleaves the N-terminus of GSDMD, inducing pyroptosis. Chemical treatments are also capable of activating caspase-3, which cleaves GSDME, initiating a GSDMD-independent pyroptosis pathway

The non-canonical pathway is a form of cell pyroptosis that does not depend on caspase-1. In sepsis, LPS in the cell walls of gram-negative bacteria is recognized and firmly bound by caspase-4/5/11. Subsequently, active caspases catalyze the production of GSDMD-NT, inducing pyroptosis [114]. Recent findings have unveiled GSDMD-independent pyroptosis mechanisms. Post-chemotherapy drug induction in GSDME-expressing cells allows caspase-3 to cleave GSDME, leading to the formation of GSDME-NT and subsequently triggering pyroptosis [115]. Moreover, granule enzymes A/B can cleave GSDMB and GSDME, respectively, stimulating their pore-forming activity and thus promoting pyroptosis [116, 117] (Fig. 4).

### Pyroptosis in ED

Excessive activation of pyroptosis has been implicated in various diabetic complications, including diabetic nephropathy and cardiomyopathy [18, 52]. Moreover, the excessive accumulation of ROS in a high-glucose environment also acts as a catalyst for the activation of the NLRP3 inflammasome [118]. Research indicated that in diabetic cardiomyopathy, one of the diabetic microvascular complications, knocking down NLRP3-related genes alleviated cardiac fibrosis and pyroptosis levels [119]. It was notable that penile corporal fibrosis was one of the primary pathological changes in DMED [15]. Interestingly, the expressions of NLRP3, ASC, caspase-1, Interleukin-1β (IL-1β), and Interleukin-18 (IL‐18) were significantly elevated in the penile corpus cavernosum tissues of DMED rats, and similar findings were observed in the detection of pyroptosis markers in penile corpus cavernosum smooth muscle cells cultured under high glucose conditions [19]. Overexpression of NLRP3 could lead to impaired nitric oxide perception in the penile corpus cavernosum smooth muscle, resulting in its relaxation dysfunction [120]. Moreover, in ED rats with a low androgen state, cell pyroptosis mediated by the NLRP3 inflammasome inhibited the rats’ erectile function [121]. Li et al. found that during the early stages of DMED progression, endothelial cell pyroptosis serves as the primary mode of cell death [122]. Recent research suggested that targeting the downregulation of NLRP3 could effectively inhibit excessive pyroptosis in the penile tissue of diabetic models, improving vascular endothelial function and smooth muscle activity and amplifying the efficacy of adipose-derived stem cell treatments for erectile function [123]. In addition, Yimusake could alleviate DMED by targeting the NLRP3-mediated pyroptosis pathway, reducing inflammation, and restoring the histological architecture and function of penile cavernous tissue [64]. Targeting pyroptosis pathways may thus represent a promising strategy for restoring erectile function in DMED.

## Conclusion

To conclude, Dysregulated PCDs—including apoptosis, autophagy, pyroptosis, and ferroptosis—play critical roles in the pathogenesis of DMED. While different PCDs exhibit distinct features, they are interconnected through mutual enhancement, conversion, and suppression. Abnormal activation of PCDs results in dysfunction and loss of key cells in the penile corpus cavernosum and initiates secondary inflammatory cascades, ultimately contributing to the development of ED.

Therapeutic strategies targeting these imbalanced PCDs, such as pharmacological agents and stem cell therapies, have shown potential in restoring erectile function. However, the underlying mechanisms of PCDs in DMED remain incompletely understood. For instance, the efficacy of drugs targeting cell death in type 2 diabetic rat models requires validation in type 1 models and vice versa. The causal relationship between cell death and oxidative stress, as well as the identification of specific upstream molecules involved in PCDs, also warrants further investigation. Moreover, there are contradictions and controversies in some of the current research findings. For instance, in the process of treating DMED by targeting autophagy, whether the therapeutic effect is produced by upregulating protective autophagy or downregulating autophagy flux remains to be further validated. Furthermore, stem cell therapy, a burgeoning modality for ED treatment, has seen certain experimental findings transition to clinical applications. Yet, its widespread adoption is hindered by ethical and safety concerns. Hence, there is a pressing need for thorough investigations of the signaling networks and the upstream and downstream signaling molecules involved in various forms of PCDs in DMED. Identifying specific targets within these pathways could provide safer and more effective therapeutic options for the prevention and treatment of DMED.

## Data Availability

No datasets were generated or analysed during the current study.

## Abbreviations

DM: Diabetes mellitus
ED: Erectile dysfunction
DMED: Diabetes mellitus-induced erectile dysfunction
PDE5is: Phosphodiesterase type 5 inhibitors
PCD: Programmed cell death
AGEs: Advanced glycation end-products
ROS: Reactive oxygen species
cGMP: Cyclic guanosine monophosphate
nNOS: Neuronal nitric oxide synthase
eNOS: Endothelial nitric oxide synthase
NO: Nitric oxide
Bcl-2: B-cell lymphoma-2
Bax: Bcl-2 associated X protein
Bak: Bcl-2 antagonist/killer
Bcl-xL: B-cell lymphoma-extra large
PTP: Permeability transition pore
Cyt-c: Cytochrome c
BH3: B-cell lymphoma-2 homology domain 3
MOMP: Mitochondrial outer membrane permeabilization
Apaf-1: Apoptotic protease activating factor-1
TNFR: Tumor necrosis factor receptor
FasR: Fatty acid synthetase receptor
FasL: Fatty acid synthetase ligand
FADD: Fas-associated death domain protein
DISC: Death inducing signaling complex
DED: Death effector domain
TRADD: TNFR1-associated death domain protein
TRAFs: TNF receptor-associated factors
RIP1: Receptor-interacting protein kinase
IAPs: Inhibitors of apoptosis proteins
NF-κB: Nuclear factor kappa-B
LCG: Leech and centipede granules
Nrf2: Nuclear factor erythroid 2-related factor 2
EMPs: Endothelial microparticles
mTOR: Mammalian target of rapamycin
AMPK: 5’AMP-activated protein kinase
ULK1: UNC-51-like kinase
ATG13: Autophagy-related proteins 13
ATG: Autophagy-related gene
AKT: Protein kinase B
VPS34: Vacuolar protein sorting 34
PAS: Pre-autophagosomal structure
PI3K: Phosphatidylinositol 3-kinase
PI3K-III: Phosphatidylinositol 3-kinase
LC3: Microtubule-associated protein 1 light chain 3
USCs: Urine-derived stem cells
ESWT: Energy shock wave therapy
MSCT: Mesenchymal stem cell therapy
MSCs: Mesenchymal stem cells
NOX1/4: NADPH oxidases1/4
LPO: Lipid peroxidation
PUFA: Polyunsaturated fatty acids
GSH: Glutathione
SLC3A2: Solute carrier family 3 member 2
SLC7A11: Solute carrier family 7a member 11
GCL: Glutamate-cysteine ligase
GSS: Glutathione synthetase
GPX4: Glutathione peroxidase 4
Tf: Tightly with transferrin
TfR1: Transferrin receptor 1
DMT1: Divalent metal transporter 1
FPN1: Ferroportin 1
NCOA4: Nuclear receptor coactivator 4
AA: Arachidonic acid
ACSL4: Acyl-CoA synthetase long-chain family member 4
LPCAT3: Lysophosphatidylcholine acyltransferase 3
LOX: Lipoxygenase
FSP1: Ferroptosis suppressor protein 1
CoQH2: Hydroquinone form of CoQ
CoQ10: Coenzyme Q10
TFRC: Transferrin receptor
NADPH: Nicotinamide adenine dinucleotide
CCSMCs: Cavenosum smooth muscle cells
GPX4-LV: GPX4 lentiviral vectors
HUCMSCs: Human umbilical cord mesenchymal stem cells
LPS: Lipopolysaccharide
PRRs: Pattern recognition receptors
ASC: Apoptosis-related speck-like protein
PYD: Pyrin domain
CARD: Caspase activation and recruitment domain
IL-1β: Interleukin-1β
IL-18: Interleukin-18
NLRP3: NOD-like receptor NLR family pyrin domain-containing
G. officinalis: Gynochthodes officinalis (F.C.How) Razafim. & B. Bremer
HO-1: Heme oxygenase-1
BaTCG: Piezoelectric barium titanate (BaTiO3) and glucagon-like peptide-1 receptor agonists (GLP-1RAs)
NEDD4: Neural precursor cell-expressed developmentally downregulated protein 4
MiRNA-100: MicroRNA-100
RhoA: Ras homolog gene family member A
ROCK: Rho-associated coiled-coil-containing kinase
Si-hmgcs2: Small interfering RNA targeting 3-hydroxy-3-methylglutaryl-CoA synthase 2
